# Germline mutations in cancer-predisposition genes in patients with biliary tract cancer

**DOI:** 10.18632/oncotarget.27224

**Published:** 2019-10-15

**Authors:** Takeshi Terashima, Kumiko Umemoto, Hideaki Takahashi, Hiroko Hosoi, Erina Takai, Shunsuke Kondo, Yasunari Sakamoto, Shuichi Mitsunaga, Izumi Ohno, Yusuke Hashimoto, Mitsuhito Sasaki, Masafumi Ikeda, Kazuaki Shimada, Shuichi Kaneko, Shinichi Yachida, Kokichi Sugano, Takuji Okusaka, Chigusa Morizane

**Affiliations:** ^1^ Department of Gastroenterology, Kanazawa University Hospital, Kanazawa, Japan; ^2^ Department of Hepatobiliary and Pancreatic Oncology, National Cancer Center Hospital, Tokyo, Japan; ^3^ Department of Hepatobiliary and Pancreatic Oncology, National Cancer Center Hospital East, Kashiwa, Japan; ^4^ Department of Cancer Genome Informatics, Graduate School of Medicine, Osaka University, Osaka, Japan; ^5^ Hepatobiliary and Pancreatic Surgery Division, National Cancer Center Hospital, Tokyo, Japan; ^6^ Oncogene Research Unit/Cancer Prevention Unit, Tochigi Cancer Center Research Institute, Tochigi, Japan; ^7^ Department of Genetic Medicine and Services, National Cancer Center Hospital, Tokyo, Japan

**Keywords:** germline mutations, biliary tract cancer, hereditary breast cancer syndrome, hereditary ovarian cancer syndrome, cancer-predisposition genes

## Abstract

The prevalence of germline mutations in patients with biliary tract carcinoma (BTC) remains unclear. Here, we investigated the prevalence and types of germline mutations in patients with BTC. We reviewed 269 patients with pathologically proven BTC and collected clinical characteristics, including medical and family histories. Additionally, we evaluated germline variants in 21 genes associated with hereditary predisposition for cancer by targeted sequencing in patients meeting ≥1 of the following criteria: 1) hereditary breast and/or ovarian cancer (HBOC) testing criteria modified for BTC, 2) Revised Bethesda Guidelines (RBGs) modified for BTC (modified RBG), 3) familial BTC criteria, or 4) young BTC criteria. Among the 269 patients, 80 met at least one criterion. Three pathogenic mutations in three patients were identified: two in *BRCA2* and one in *BRCA1*. Among the 16 patients meeting modified HBOC testing criteria, 2 harbored germline *BRCA2* mutations, and 1 harbored a germline *BRCA1* mutation. However, no mutation in mismatch-repair genes were detected, despite 63 patients meeting modified RBG screening criteria and 18 qualifying as young BTC patients. We detected high prevalence of pathogenic germline mutations in *BRCA1/2* and none in mismatch-repair genes in BTC patients following enrichment according to family or medical history in this study.

## INTRODUCTION

The incidence of biliary tract carcinoma (BTC) is high in eastern Asia and continues to increase worldwide [1]. Patients with BTC have unsatisfactory outcomes, because the majority of them present with advanced and unresectable disease [2], with most patients subsequently developing recurrence, even after curative surgery [3]. Although combination chemotherapy with gemcitabine and cisplatin has become the standard care for patients with unresectable or recurrent BTC, its efficacy is limited, with an overall survival of <1 year [4, 5]. Therefore, BTC represents a major health concern. Several factors, including hepatolithiasis, parasitic infections, as typified by *Opisthorchis viverrini* which is unusual in Japan, primary sclerosing cholangitis, and specific toxins, have been identified as risk factors associated with BTC [6]; however, there are no reports regarding the frequency of germline mutations in cancer-predisposition genes in BTC.

Recently, knowledge concerning hereditary cancer syndrome has progressed, with sporadic studies reporting that BTC is caused by germline mutations in DNA mismatch-repair (MMR) genes, such as *MLH1*, *MSH2*, *MSH6*, and *PMS2*, capable of inducing Lynch syndrome [7]. Other germline mutations, such as *BRCA1* and *BRCA2*, which are known to be the respective causes of hereditary breast and/or ovarian cancer (HBOC) syndrome, were also identified in patients with BTC [8, 9]. Thus, a certain proportion of BTC must be caused by germline mutations in these cancer-predisposition genes; however, no systematic investigation concerning these hereditary cancer syndromes in BTC has been conducted, and their accurate prevalence remains unclear. Moreover, we hypothesized that young BTC patients would have characteristic genetic backgrounds [10]; however, information regarding this hypothesis is scarce. Here, we performed targeted sequencing of 21 representative genes related to hereditary cancer to investigate the prevalence of germline mutations in BTC patients.

## RESULTS

### Patient characteristics

**Figure 1:**
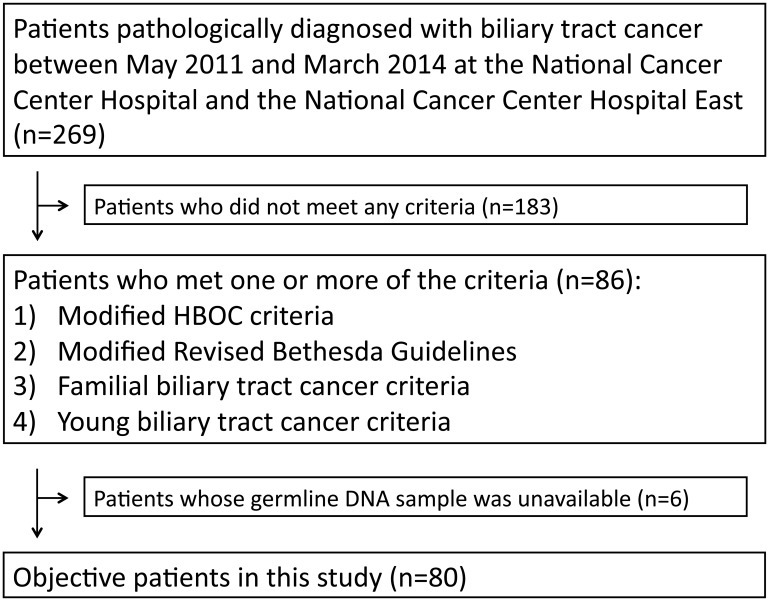
Number of patients meeting criteria for inclusion in the HBOC group, Lynch group, Familial BTC group, and Young BTC group, respectively.

Between May 2011 and March 2014, 269 patients were identified as having been pathologically diagnosed with BTC at the National Cancer Center Hospital and the National Cancer Center Hospital East. The patient characteristics are summarized in Table 1. The primary organs of the cancers included the intrahepatic bile duct (IHBD), extrahepatic bile duct (EHBD), gall bladder, and ampulla of Vater in 28.3%, 39.4%, 24.5%, and 7.8% of patients, respectively, which was consistent with a previous report [11]. Sixteen patients (5.9%), 63 patients (23.4%), 13 patients (4.8%), and 18 patients (6.7%) met the criteria for entry into the HBOC group, Lynch group, Familial BTC group, and Young BTC group, respectively. Because the germline DNA samples of six patients were unavailable, a total of 80 patients met at least one criterion and analyzed in this study (Figure 1).

**Table 1:** Patients characteristics

	Overall (*n* = 269)	Analyzed patients in this study (*n* = 80)	HBOC group (*n* = 16)	Lynch group (*n* = 63)	Familial BTC group (*n* = 13)	Young BTC group (*n* = 18)
Age, years						
Median	70	66	66	67	64	48
Range	26–90	26–90	51–78	26–90	45–73	26–50
Gender, n (%)						
Male	180 (66.9)	55 (68.8)	11 (68.8)	41 (34.9)	11 (84.6)	11 (61.1)
Female	89 (33.1)	25 (31.2)	5 (31.3)	22 (65.1)	2 (15.4)	7 (38.9)
Primary organ, n (%)						
Intrahepatic bile duct	76 (28.3)	25 (31.3)	3 (18.8)	22 (34.9)	4 (30.8)	9 (50.0)
Extrahepatic bile duct	106 (39.4)	25 (31.3)	7 (43.8)	17 (27.0)	6 (46.2)	2 (11.1)
Gallbladder	66 (24.5)	24 (30.0)	5 (31.3)	19 (30.2)	2 (15.4)	6 (33.3)
Ampulla of Vater	21 (7.8)	6 (7.5)	1 (6.3)	5 (7.9)	1 (7.7)	1 (5.6)
Body mass index						
Median	22.0	22.0	21.6	22.0	24.1	23.6
Range	14.8-33.0	16.6–33.0	16.6–28.0	17.0–33.0	18.4–28.1	17.0–33.0
Smoking Index, n (%)						
<400	159 (59.1)	35 (43.8)	8 (50.0)	37 (58.7)	7 (53.8)	14 (77.8)
≥400	110 (40.9)	45 (56.3)	8 (50.0)	26 (41.3)	6 (46.2)	4 (22.2)

### Analysis of germline variants

Targeted sequencing showed that the patients harbored a median of 22 variants (range: 15–32) in the 21 targeted genes. Of these, three variants in three patients were considered pathogenic (Table 2), with the clinicopathologic characteristics of the patients shown in Table 3. Additionally, 57 variants in 50 patients were considered variants of unknown significance (VUS), with the clinicopathologic characteristics of these patients summarized in Supplementary Table 1.

**Table 2:** Germline truncating mutation found in this study

Patient ID	Gene	Reference sequence	dbSNP Accession Number	Nucleotide change	Amino acid change	Type of mutation	dbSNP	ClinVar	PolyPhen2	MutationTaster	FATHMM	Our interpretation for pathogenicity
19	*BRCA2*	NM_000059	Rs397507568	c.10150 C>T	p. Arg3384Ter	Nonsense	VUS	Conflicting	—	Disease causing	—	VUS
31	*BRCA1*	NM_007294	rs80356923	c.3640 G>T	p. Glu1214Ter	Nonsense	Other	Pathogenic	—	Disease causing	Cancer	Pathogenic
37	*BRCA2*	NM_000059	rs80359520	c.5574_5577 delAATT	p. Ile1859Lys fs*3	Frameshift	Pathogenic	Pathogenic	—	Disease causing	—	Pathogenic
57	*BRCA2*	NM_000059	rs80359314	c.1887_1888 insA	p. Thr630Asn fs*6	Frameshift	Pathogenic	Pathogenic	—	Disease causing	—	Pathogenic

**Table 3:** Clinicopathological characteristics of the patients with germline truncating mutation

Patient ID	Gene	Previous cancer	Cancer in first- or second- degrees relatives	Age at onset, years	Sex	Primary organ of tumor *
19	*BRCA2*	None	1 breast, 1 lung, and 1 esophageal	65	M	Gallbladder
31	*BRCA1*	None	1 ovarian and 1 uterus	65	M	EHBD
37	*BRCA2*	None	1 prostate, 1 biliary, 1 uterus, and 1 colorectal	57	M	IHBD
57	*BRCA2*	None	1 breast	55	F	IHBD

* EHBD: extrahepatic bile duct, IHBD: intrahepatic bile duct.

The *BRCA1* variant c.3640G>T (p. Glu1214Ter) was detected in a male patient with EHBD cancer diagnosed at 65-years old. The patient had no history of malignant disease and had a family history of ovarian cancer diagnosed in a sister at 55-years old. The mutation was reportedly detected in the affected members from one family with ovarian cancer and a patient with breast cancer, but not in a healthy woman [12]. Another report described the mutation as detected in one family among 643 Dutch and 23 Belgian HBOC families [13]. Therefore, we classified the variants as pathogenic.

Two other *BRCA2* variants, c.5574_5577 delAATT (p. Ile1859Lys fsX3) and c.1887_1888 insA (p. Thr630Asn fsX6), resulting in frameshift mutations were detected in a male patient with IHBD cancer diagnosed at 57-years old and in a female patient with IHBD cancer diagnosed at 55-years old, respectively. They had no history of malignant disease; however, the former patient had a family history of metachronous cancers of the biliary tract and prostate cancer in the father and lung cancer in the mother, and the later patient had a family history of breast cancer diagnosed in an aunt at 30-years old. There were no studies reporting clinical significance or functional effects of the latter variant, whereas the former mutation was found in at least three families, two of which were kindred with more than three instances of breast cancer, suggesting it as the most frequent pathogenic variant in China [14]. Considering the effects of the former variant on the protein and interpretations as pathogenic according to dbSNP, we also classified this variant as pathogenic.

**Figure 2:**
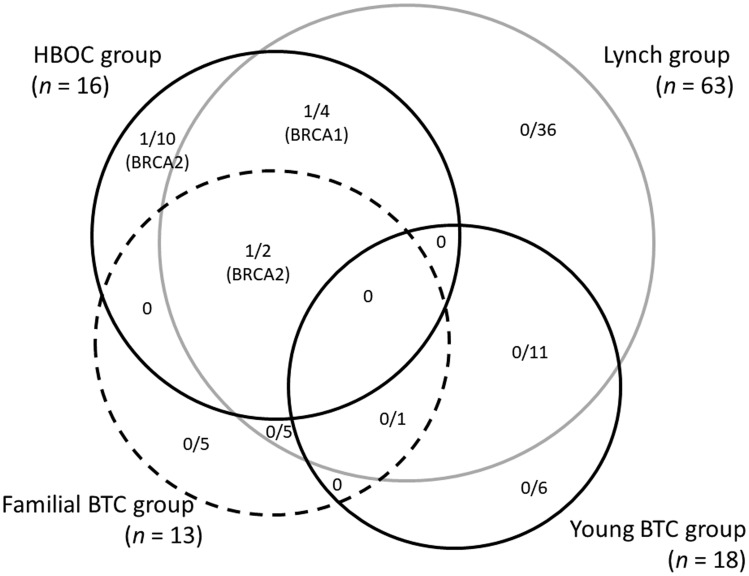
The prevalence and types of germline mutations classified as pathogenic according to the groups used in this study.

The prevalence and types of germline mutations classified as pathogenic according to the groupings used in this study are summarized in Figure 2. Three of 16 patients (18.8%), 20 of 63 patients (3.2%), 1 of 13 patients (7.7%), and none of 18 patients harbored pathogenic variants in the HBOC group, Lynch group, Familial BTC group, and Young BTC group, respectively. All 3 pathogenic variants detected in the HBOC group were *BRCA* genes whereas no mutation in mismatch-repair genes were detected despite 63 patients meeting modified RBG screening criteria. In regard to primary organs, 2 of 76 IHBD cancer patients (2.6%), 1 of 106 EHBD cancer patient (0.9%), and no gall bladder cancer and ampulla of Vater patients were founded to have pathogenic variants.

## DISCUSSION

Given the lack of studies investigating the frequency of germline mutations in cancer-predisposition genes associated with BTC, the importance of hereditary cancer syndromes in BTC remains unclear. Although insufficient attention has been given to the impact of family or medical history of cancers in relation to BTC-patient medical care, we hypothesized that proportions of BTC patients with a family history of cancer and/or young-onset BTC patients would harbor germline mutations in cancer-predisposition genes, similar to colorectal cancer (CRC), ovarian cancer, and breast cancer. To select patients for genetic investigation, we referred to previously established testing criteria for HBOC, Lynch syndrome, and familial pancreatic cancer and modified them for suitability to BTC for this study. We subsequently performed targeted gene sequencing in patients with BTC to investigate the prevalence and types of germline mutations.

The most important insight gained from our results was that up to 19% of the patients enriched according to modified HBOC testing criteria harbored pathogenic variants in *BRCA1*/*2. BRCA1*/*2* is involved in maintenance of genome stability, and inherited mutations in these genes increase lifetime risk of developing HBOC-related cancers [15, 16]. Germline mutations in *BRCA1/2* or other HBOC-related genes are tested to determine whether the patients have a medical or family history of breast or/and ovarian cancer, with the prevalence of these mutations at ~25% in patients with breast or ovarian cancer [17]. Here, *BRCA1/2* variants were detected in a higher proportion of the patients than our expectation following adoption of the modified HBOC testing criteria.

We identified no *MSH6*, *MLH1*, *MSH2*, or *PMS2* variants classified as pathogenic in BTC patients, despite enrichment according to modified RBGs focused on BTC instead of CRC. BTC is known to be one of Lynch syndrome-related tumors, and several clinical and case reports regarding Lynch syndrome-related BTC patients exist [7]. However, we could not find any patients in our study harboring germline mutation(s) of MMR genes contrary to our expectation. Recently universal tumor screening has been proposed for all colorectal and endometrial cancers [18, 19], and we cannot deny the usefulness of the widely accepted criteria as first-line screening criteria for Lynch syndrome; however, a previous study reported its exhibiting low specificity [20]; therefore, it might be necessary to establish other methods to enrich criteria associated with Lynch syndrome-related BTC patients. Similarly, specific genetic mutations were limited in the Familial and Young BTC groups among the 21 genes. In future work, whole-genome sequencing should be performed to investigate the genetic background of this cohort.

We did not determine the fourth BRCA2 variant found in this study, c.10150 C>T (p. Arg3384Ter), as pathogenic in consequence of sufficient for discussion because the variant located in the final exon and CIMBA criteria excluded the variants which truncate after codon 3326 which are currently considered neutral. However, the mutation was reported to detect among patients with unilateral breast cancer [21] and in a Korean patient with hereditary breast cancer [22]. This patient also had family histories of breast, lung, and esophagus. Considering this nonsense mutations causing truncation of the protein, additional studies will be needed to confirm our interpretation.

Increasing our understanding of the prevalence or types of germline mutations in patients with BTC benefits the development of improved screening methods for BTC or related cancers for patients and their families. Moreover, identification of these mutations offers more effective treatment possibilities for individual patients. Specific agents, such as platinum compounds or poly (ADP-ribose) polymerase (PARP) inhibitors, are effective treatments for some cancers involving *BRCA1* or *BRCA2* mutation [23–25]. Our results showing high prevalence of *BRCA1* or *BRCA2* variants in BTC patients also suggested BTC as a good target for the development of platinum or PARP-inhibitor therapeutics.

There were limitations to our study. First, we underestimated the impact of medical or family history of cancer due to the retrospective nature of the study. Second, we used a conservative approach to classifying the variants. At last, objective patients were restricted to Japanese. Therefore, further study is required to validate our findings.

In conclusion, up to 19% of the BTC patients in this study harbored pathogenic variants in *BRCA1* and *BRCA2* following patient classification according to modified HBOC testing criteria. No germline mutation in MMR genes was observed among following patient classification according to modified Revised Bethesda Guidelines in our study.

## MATERIALS AND METHODS

### Patients

Objective patients in this study were those with pathologically proven BTC, including cancers of the IHBD, EHBD, gall bladder, and ampulla of Vater, between May 2011 and March 2014 at the National Cancer Center Hospital (Tokyo, Japan) and the National Cancer Center Hospital East (Kashiwa, Japan). We reviewed the medical records of these patients and collected the following clinicopathologic features, as well as medical and family histories of any cancer in all relatives: age at onset, gender, body mass index, smoking history, and primary organ of tumor. The institutional Review Board at the National Cancer Center approved this study, which was conducted in accordance with the Declaration of Helsinki and Japanese national regulations, as well as the Ethical Guidelines for Medical and Health Research Involving Human Subjects (available: https://www.mhlw.go.jp/file/06-Seisakujouhou-10600000-Daijinkanboukouseikagakuka/0000080278.pdf). Each patient provided informed consent.

### Criteria for targeted gene sequencing

Targeted gene sequencing was performed on samples from patients suspected of harboring any germline mutations based on their meeting one or more of the following criteria: 1) modified HBOC criteria which was based on *BRCA1/2* testing criteria outlined by the National Comprehensive Cancer Network Clinical Practice Guidelines in Oncology Genetic/Familial High-Risk Assessment version 2.2017 and modified by the addition of BTC as well as pancreatic and prostate cancer (HBOC group; Supplementary Table 2); 2) modified Revised Bethesda Guidelines (RBGs) which was based on RBGs for hereditary nonpolyposis CRC [26] modified by the addition of personal or family history within second-degree relatives of BTC to CRC (Lynch group; Supplementary Table 3); 3) familial BTC criteria; a patient with at least one first-degree relatives with a history of BTC (Familial BTC group); and 4) young BTC criteria; a patient diagnosed with BTC at a young age (≤50-years old; Young BTC group).

### Massive parallel sequencing of target genes

Germline DNA samples were extracted from peripheral-blood leukocytes provided by the National Cancer Center Biobank, Japan. A custom targeted-capture kit was designed using NimbleDesign (NimbleGen, Madison, WI, USA) targeting the exons and splice-sites of 21 genes associated with hereditary predisposition for cancer (*BRCA1, BRCA2, CHEK2, PALB2, ATM, BRIP1, TP53, PTEN, STK11, CDH1, NBN, BARD1, MLH1, MRE11, MSH2, MSH6, MUTYH, PMS1, PMS2, RAD50,* and *RAD51C*). Sequencing libraries were created using the SeqCaq EZ Library (NimbleGen) and KAPA Library Preparation Kits (Kapa Biosystems, Wilmington, MA, USA) according to manufacturer protocol. Targeted-capture sequencing was performed on Illumina HiSeq2500 platforms (Illumina, San Diego, CA, USA). With the intent to maximize the sensitivity of variant detection, no variant-quality filters were applied. Bases were called using Illumina BCLFAST2 (Illumina). Paired-end reads were aligned to the human reference genome (GRCh37) using the Burrows-Wheeler Aligner [27]. A Genome Analysis Toolkit (GATK) was used to detect single-nucleotide substitutions and small insertions and deletions using best practices derived from the GATK website (https://www.broadinstitute.org/gatk/) [28].

### Variant characterization

Variants in 21 genes were considered for analysis if they met one of the following criteria: 1) a non-reference call by GATK; 2) predicted to affect the protein sequence or splice site (i. e., ±5 base pairs); and 3) a genotypic frequency of <1% in the 1,000 Genomes Project [29, 30], dbSNP [31], the Exome Aggregation Consortium (https://www.broadinstitute.org/gatk/), or the Japanese Human Genetic Variation Browser (http://www.genome.med.kyoto-u.ac.jp/SnpDB/) and the Integrative Japanese Genome Variation Database (https://ijgvd.megabank.tohoku.ac.jp/). These rare, non-synonymous variants were classified as either pathogenic, benign, or VUS. Prediction by dbSNP or ClinVar (https://www.ncbi.nlm.nih.gov/clinvar) was used as a reference for our classification. For *CDH1*, *MLH1*, *MSH2*, *MSH6*, *MUTYH*, *PMS1*, and *PMS2*, variants were classified according to the InSiGHT consortium (https://www.insight-group.org/variants/databases/) [32]. For *BRCA1* and *BRCA2*, variants were classified using the database generated by Vallee et al. [33] and assessed using the Leiden Open Variation Database (LOVD) (http://hci-exlovd.hci.utah.edu/home.php).

Rare, non-synonymous variants not found in these databases were classified based on their predicted effect on the protein product. Nonsense variants and variants changing the canonical splice sites (i. e., ±2 base pairs), as well as frameshift insertions and deletions, were considered pathogenic unless they occurred in the final exon. As for identification of functional missense mutations, SIFT (http://sift.jcvi.org) [34], Polyphen-2 (http://genetics.bwh.harvard.edu/pph2/) [35], MutationTaster (http://www.mutationtaster.org) [36], and Functional Analysis through Hidden Markov Models (http://fathmm.biocompute.org.uk) [37] were employed, as well as for a literature review. Classification of each VUS or pathogenic variant was determined by our medical genetics team, including two clinical geneticists in our hospital.

### Sanger sequencing

Variants classified as deleterious or likely deleterious in targeted-capture sequencing were validated by Sanger sequencing. Polymerase chain reaction (PCR) amplification was performed using 20 ng of genomic DNA with intronic primers flanking targeted exons. PCR products were sequenced using the M13F primer (5′-GTAAAACGACGGCCAGT-3′) or the M13R primer (5′-CAGGAAACAGCTATGACC-3′) incorporated into the forward and reverse primers of each primer pair, respectively. These results were analyzed with Sequencher 5.0.1 software (Gene Codes, Ann Arbor, MI, USA).

## SUPPLEMENTARY MATERIALS





## Abbreviations

BTC: biliary tract carcinoma
HBOC: hereditary breast and/or ovarian cancer
RBG: Revised Bethesda Guideline
MMR: mismatch-repair
IHBD: intrahepatic bile duct
EHBD: extrahepatic bile duct
VUS: variants of unknown significance
CRC: colorectal cancer
PARP: poly(ADP-ribose) polymerase
PCR: polymerase chain reaction
