# Supplementary material for: Germline mutations in cancer-predisposition genes in patients with biliary tract cancer
Source: Oncotarget. 2019 Oct 15;10(57):5949–57. doi: 10.18632/oncotarget.27224 (PMC6800267; doi:10.18632/oncotarget.27224)
Supplement: Supplementary file 1 [file oncotarget-10-5949-s001.pdf]

Germline mutations in cancer-predisposition genes in patients with biliary tract cancer

SUPPLEMENTARY MATERIALS

Supplementary Table 1: Germline mutation classified as benign or variants of unknown significance and clinical characteristics of the patients with those mutations in this study. See Supplementary Table 1

Supplementary Table 2: Inclusion criteria for hereditary breast and/or ovarian cancer related cancer used in this study

- Individual from a family with a known deleterious *BRCA1/BRCA2* gene mutation
- Personal history of breast cancer + one or more of the following:
    - » Diagnosed ≤45 years old
    - » Diagnosed ≤50 years old with:
        - An additional breast cancer primary
        - ≥1 close blood relative with breast cancer at any age
        - ≥1 close blood relative with pancreatic cancer
        - ≥1 relative with prostate cancer (Gleason score ≥7)
        - ≥1 relative with biliary tract cancer
        - An unknown of limited family history
    - » Diagnosed ≤60 years old with:
        - Triple negative breast cancer
    - » Diagnosed at any age with:
        - ≥2 close blood relatives with breast cancer, pancreatic cancer, prostate cancer (Gleason score ≥7), or biliary tract cancer at any age
        - ≥1 close blood relative with breast cancer diagnosed ≤50 years old
        - ≥1 close blood relative with ovarian carcinoma
        - A close male blood relative with breast cancer
        - For an individual of ethnicity associated with higher mutation frequency no additional family history may be required
- Personal history of ovarian carcinoma
- Personal history of male breast cancer
- Personal history of prostate cancer (Gleason score ≥7) at any age with ≥1 close blood relative with ovarian carcinoma at any age or breast cancer diagnosed ≤50 years old or two relatives with breast, pancreatic, prostate cancer (Gleason score ≥7), or biliary tract cancer at any age
- Personal history of pancreatic cancer at any age with ≥1 close blood relative with ovarian carcinoma at any age or breast cancer diagnosed ≤50 years old or two relatives with breast, pancreatic, prostate cancer (Gleason score ≥7), or biliary tract cancer at any age
- Personal history of biliary tract cancer at any age with ≥1 close blood relative with ovarian carcinoma at any age or breast cancer diagnosed ≤50 years old or two relatives with breast, pancreatic, prostate cancer (Gleason score ≥7), or biliary tract cancer at any age
- Personal history of pancreatic cancer and Ashkenazi Jewish ancestry
- *BRCA1/2* mutation detected by tumor profiling in the absence of germline mutation analysis
- Family history only (significant limitations of interpreting test results for an unaffected individual should be discussed):
    - » First- or second-degree blood relative meeting any of the above criteria
    - » Third-degree blood relative who has breast cancer and/or ovarian carcinoma and who has ≥2 close blood relatives with breast cancer (at least one with breast cancer diagnosed ≤50 years old) and/or ovarian carcinoma

Supplementary Table 3: Inclusion criteria for Lynch syndrome related cancer used in this study

- Colorectal cancer or biliary tract cancer diagnosed in a patient who is less than 50 years of age.
- Presence of synchronous, metachronous colorectal, or other hereditary nonpolyposis colorectal cancer-associated tumors, regardless of age.
- Colorectal cancer or biliary tract cancer with the microsatellite instability-high histology diagnosed in a patient who is less than 60 years of age.
- Colorectal cancer or biliary tract cancer diagnosed in one or more first-degree relatives with a hereditary nonpolyposis colorectal cancer-associated tumors, with one of the cancers being diagnosed under age 50 years.
- Colorectal cancer or biliary tract cancer diagnosed in two or more first- or second-degrees relatives with hereditary nonpolyposis colorectal cancer-associated tumors, regardless of age.
